# Immune horses rapidly increase antileukoproteinase and lack type I interferon secretion during mucosal innate immune responses against equine herpesvirus type 1

**DOI:** 10.1128/spectrum.01092-24

**Published:** 2024-08-20

**Authors:** Camille M. Holmes, Susanna Babasyan, Naya Eady, Christiane L. Schnabel, Bettina Wagner

**Affiliations:** 1 Department of Population Medicine and Diagnostic Sciences, College of Veterinary Medicine, Cornell University, Ithaca, New York, USA; 2 Biotechnological-Biomedical Center, Leipzig University, Leipzig, Germany; Michigan State University, East Lansing, Michigan, USA

**Keywords:** equine herpesvirus, mucosal immunity, innate immune response

## Abstract

Equine herpesvirus type 1 (EHV-1) is one of the most prevalent respiratory pathogens in horses with a high impact on animal health worldwide. Entry of the virus into epithelial cells of the upper respiratory tract and rapid local viral replication is followed by infection of local lymphoid tissues leading to cell-associated viremia and disease progression. Pre-existing mucosal immunity has previously been shown to reduce viral shedding and prevent viremia, consequently limiting severe disease manifestations. Here, nasopharyngeal transcriptomic profiling was used to identify differentially expressed genes following EHV-1 challenge in horses with different EHV-1 immune statuses. Immune horses (*n* = 4) did neither develop clinical disease nor viremia and did not shed virus after experimental infection, while non-immune horses (*n* = 4) did all the above. RNA sequencing was performed on nasopharyngeal samples pre- and 24 hours post-infection (24hpi). At 24hpi, 109 and 44 genes were upregulated in immune horses and non-immune horses, respectively, and three genes were explored in further detail. Antileukoproteinase (*SLPI*) gene expression increased 2.1-fold within 24 hours in immune horses in concert with protein secretion. Interferon (IFN)-induced proteins with tetratricopeptide repeats 2 (*IFIT2*) and 3 (*IFIT3*) were upregulated in non-immune horses, corresponding with nasal IFN-α secretion and viral replication. By contrast, neither *IFIT* expression nor IFN-α secretion was induced by EHV-1 infection of immune horses. Transcriptomic profiling offered a tool to identify, for the first time, the role of SLPI in innate immunity against EHV-1, and further emphasized the central role of the type I IFN response in the anti-viral defense of non-immune horses.

**IMPORTANCE:**

Equine herpesvirus type 1 (EHV-1) remains a considerable concern in the equine industry, with yearly outbreaks resulting in morbidity, mortality, and economic losses. In addition to its importance in equine health, EHV-1 is a respiratory pathogen and an alphaherpesvirus, and it may serve as a model for other viruses with similar pathogenicity or phylogeny. Large animal models allow the collection of high-volume samples longitudinally, permitting in-depth investigation of immunological processes. This study was performed on bio-banked nasopharyngeal samples from an EHV-1 infection experiment, where clinical outcomes had previously been determined. Matched nucleic acid and protein samples throughout infection permitted longitudinal quantification of the protein or related proteins of selected differentially expressed genes detected during the transcriptomic screen. The results of this manuscript identified novel innate immune pathways of the upper respiratory tract during the first 24 hours of EHV-1 infection, offering a first look at the components of early mucosal immunity that are indicative of protection.

## INTRODUCTION

The mucosal immune system is the first line of defense against external pathogens and consists of heterogeneous populations of immune cells within the epithelium and underlying mucosal-associated lymphoid tissues (MALT). The upper respiratory tract (URT) and lower respiratory tract (LRT) represent unique mucosal barriers that face a variety of environmental exposures under homeostatic conditions, and during respiratory infection, they are the primary sites of viral entry and replication. In humans, a pro-inflammatory type I interferon response drives the anti-viral defense in the respiratory tract, which regulates the activation of resident immune cells or recruitment from the periphery (1). Recent transcriptomic profiling of primary human respiratory epithelial cells emphasized their role in orchestrating the immune response through the production of a wide array of cytokines and anti-microbial peptides upon toll-like receptor (TLR) stimulation (2). Localized mucosal immune cells in the respiratory tract can contribute to viral clearance through various mechanisms including secretion of neutralizing antibodies by plasma cells (3, 4), T and NK cell-mediated cytotoxicity (5, 6), and coordination and regulation of immune responses by monocyte-derived cell populations (7, 8). Together, the components of the mucosal immune response at the epithelial barriers drive the initial defense against viral pathogens and can, upon successful activation and regulation, prevent the development of severe disease.

Equine herpesvirus type I (EHV-1) is an alphaherpesvirus endemic in horse populations worldwide. EHV-1 infects horses through the URT and like other herpesviruses can establish latency within its host (9). Reactivation commonly occurs during periods of stress, including parturition, which allows for early life vertical transfer from mare to foal (10). Weaning, transport to a new facility, or changes in herd dynamics also promote EHV-1 reactivation, with viral transmission occurring *via* direct nose-to-nose contact with an infected horse or by fomites (11–14). Immune horses clear the virus from the respiratory tract within a few days of exposure, preventing clinical manifestations (15, 16). Non-immune horses develop fever, mild to moderate respiratory disease, and establish cell-associated viremia in peripheral blood leukocytes before clearing the virus within 10–20 days. In some horses, EHV-1 can spread during viremia to the pregnant uterus or the central nervous system (CNS) (10, 13, 14, 17, 18). In these cases of EHV-1 infection, viral replication in vascular endothelial cells can lead to local thrombosis and ischemia (19), manifesting as equine herpesvirus myeloencephalopathy (EHM) in the CNS and/or abortion in pregnant mares (11, 20–23). This highlights the importance of a rapid and protective immune response at the mucosal entry site, reducing cell-associated viremia and preventing severe disease outcomes.

The mucosal immune response to EHV-1 was first investigated in the respiratory tract, where viral proliferation and degradation of host tissue are seen within the first week of infection (24). Characterization of immune cell populations in the lung showed an increase in the CD8^+^ T cell population and a corresponding drop in the CD4^+^ T cell population of non-immune horses, while minor changes in T cell populations were observed in immune horses (25). Mucosal EHV-1-specific T cells were found both within the lung (25) and later in the MALT of EHV-1-infected horses (26). More recent work has emphasized the production of cytokines and EHV-1-specific antibodies at the nasal mucosa of the URT. Immune horses have pre-existing mucosal antibodies and significantly increase anti-EHV-1 IgG4/7 secretion at the respiratory entry site within 1 day of viral exposure (15, 16). Mucosal IgG4/7 antibodies can effectively neutralize the virus at the site of entry, prevent complete viral replication, and are thus instrumental in preventing viral entry at the URT and subsequent disease (27). By contrast, non-immune horses slowly develop a mixed response of IgG1 and IgG4/7 by 8 and 10 days post-infection, respectively (15, 16, 28, 29). The timing and magnitude of mucosal pro-inflammatory and chemotactic cytokine secretion is also dependent on the immune status of the horse. Nasal secretion of interferon-alpha (IFN-α), c-c motif chemokine ligand 2 (CCL2), CCL3, CCL5, CCL11, secretory cluster of differentiation 14 (sCD14), interferon-gamma (IFN-γ), and interleukin 10 (IL-10) is detectable in nasal secretions of non-immune horses following viral infection (15, 16, 28, 29), but only anti-inflammatory IL-10 and the T cell chemokine CCL5 are secreted in immune horses (15, 16). While both IFN-α and IFN-γ responses occur in non-immune horses, type I IFN-α secretion starts earlier, is of higher magnitude, and is a dominating factor driving the inflammatory response at the nasal mucosa (16), as mentioned above for human respiratory viral infections (1).

In addition, nasal explant models have been used to study the anti-viral responses against EHV-1 at the mucosa of the URT *in vitro*. These models focused primarily on pathogenesis including viral replication kinetics (30, 31), mucosal damage (32), and infection of leukocytes (33, 34). The models suggest rapid viral infiltration through the basement membrane within 36 hours of infection, which is facilitated by infection of leukocytes (30). Further characterization of the infected cells identified mucosal CD172^+^ cells which readily migrated through the epithelium with CCL2 and CCL5 acting as drivers of the migration (34), possibly facilitating viral transmission through the epithelium (33). *In vivo* experiments agree with some aspects of the nasal explant models, such as increased mucosal CCL2 and CCL5 secretion during infection. However, nasal explant models cannot fully recapitulate the complexity of the heterogeneous cell populations and the contributions of localizing lymphocytes at the mucosa during the mucosal immune response to EHV-1. Nasal explant models thus have great value for discovering viral processes but are rather restricted for evaluating a productive and complex mucosal host immune response.

With the availability of a horse reference genome (35), transcriptomic profiling offers a platform to broadly investigate the regulation of host responses upon EHV-1 infection including immune relevant pathways. Samples can be obtained by non-invasive methods to monitor changes in the immune response over time within a single or multiple individuals. RNA sequencing (RNAseq) has previously been utilized to quantify mRNA expression in human airway diseases, with samples collected by swabbing the nasal cavity using a cytology brush in a brief outpatient procedure (36, 37). RNAseq has also been used in the context of EHV-1, to characterize gene expression in peripheral blood mononuclear cells (PBMC) following infection. All horses in the latter study were non-immune and the PBMC upregulated genes involved in the interferon response pathway at the peak of viremia compared to pre-infection (38), which agrees with the previously described increase in serum IFN-α in non-immune, EHV-1 infected horses (16). To analyze mucosal immune responses against EHV-1, nasopharyngeal swabs can be utilized to capture the soluble and cellular content of the nasal lumen. The nasal cells captured by the swabs include both structural epithelial cells and immune cells. Here, we compared changes in equine RNA expression at the nasal mucosa between immune and non-immune horses. Nasopharyngeal swab samples from both groups were analyzed prior to and 1 day following experimental EHV-1 infection to explore early differences in innate mucosal immunity in the context of the immune status of the horses.

## RESULTS

### Host gene expression at the mucosal entry site of EHV-1-infected horses

**Fig 1:**
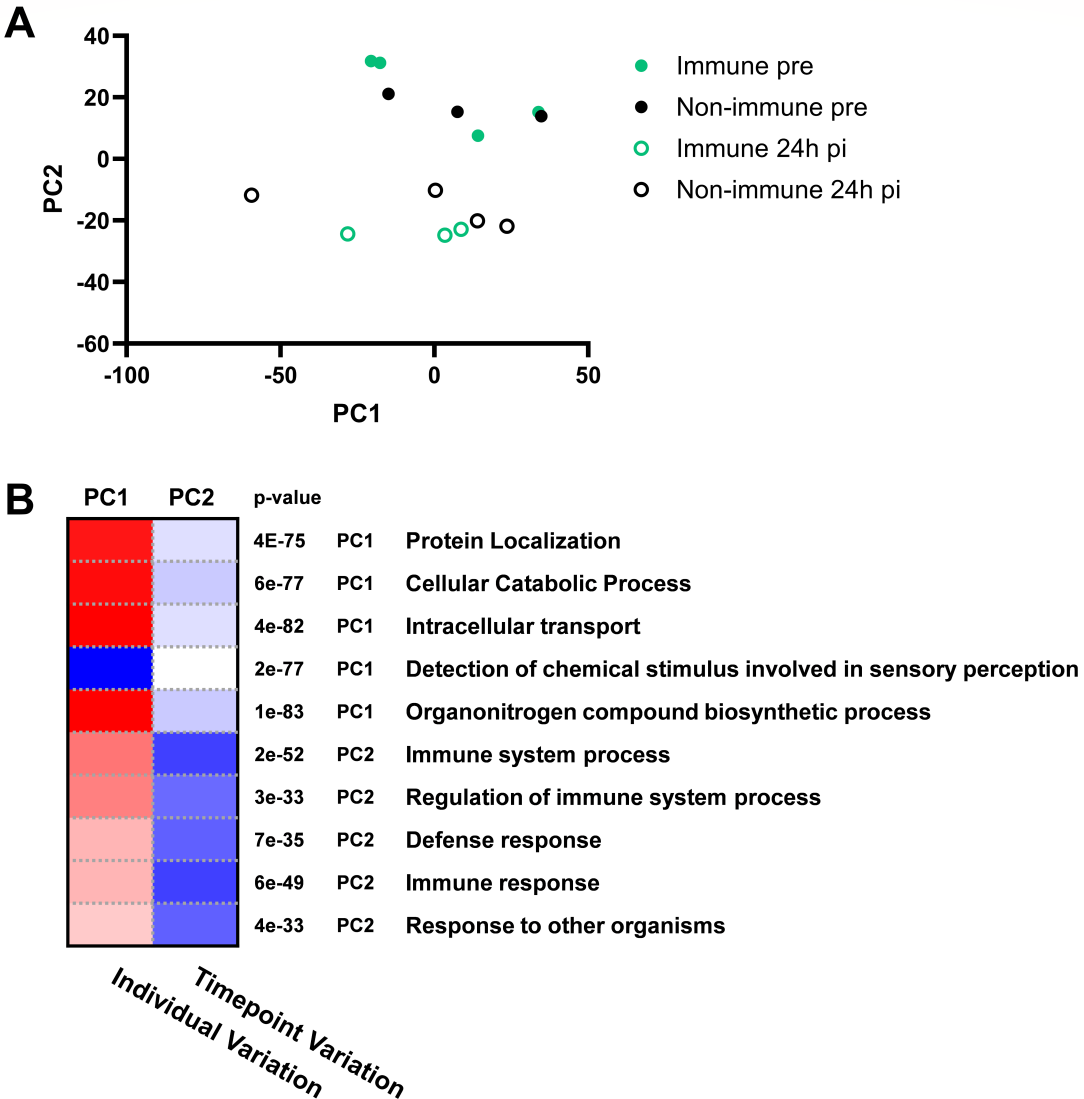
Sample variation by PCA. EHV-1 immune (*n* = 4) and non-immune (*n* = 4) horses were intranasally challenged with 1 × 10^7^ PFU of EHV-1 Ab4. RNA was extracted from nasopharyngeal swab samples, pre-infection (pre) and at 24hpi, and sequenced. Raw read counts were variance stabilizing transformation transformed, then linearly transformed by PCA for two-dimensional scaling of sample-to-sample variation. (**A**) PCA plot graphed on PC1 and PC2. Immune horses are shown in green and non-immune horses are shown in black. Filled circles represent the pre-infection timepoint and unfilled points represent 24hpi. (**B**) Pathway analysis of PCA rotation, displaying gene ontology biological process (GO:BP) pathways for PC1 (individual variation) and PC2 (pre- to 24hpi changes in gene expression). Upregulated pathways are shown in blue and downregulated pathways in red. The significance of pathway enrichment based on PC1 or PC2 is described by *P*-values.

The URT is the primary site of viral entry and replication during EHV-1 infection. To assess early changes in the host mucosal immune response, horses were experimentally infected with the neuropathogenic EHV-1 strain Ab4. Following resolution from infection, horses were retrospectively classified as non-immune based on nasal shedding of the virus, cell-associated viremia, and fever, or immune when they developed none of these viral or clinical parameters (Table 1). Nasopharyngeal samples from four non-immune and four immune horses collected before infection and at 24hpi were analyzed by RNAseq. Differential expression analysis was conducted, and principal component analysis (PCA) separated pre-infection from 24hpi timepoints by principal component 2 (PC2). PC1 did not distribute based on immune status or timepoints and represents the natural variation between individuals (Fig. 1A). Pathways upregulated within the PC2 grouping corresponded to genes upregulated from pre to 24hpi (Fig. 1A) agreeing with the expected stimulation of the immune response upon viral infection (Fig. 1B).

**TABLE 1:** EHV-1 immune status based on clinical evaluation of horses and viral detection^*f*^^,^^*g*^

Horse information^*a*^		Highest body temperature^*b*^		Highest nasal shedding^*c*^		Highest viremia^*d*^		Intranasal EHV-1-specific IgG4/7^*e*^	
EHV-1 Immune status	ID	°C	Hour post-infection	PFU/mL	Day post-infection	Ct	Day post- Infection	pre- MFI	24hpi MFI
Non-immune	Horse 3	41.2	36	19,500	3	31.25	6	2	2
	Horse 5	42.4	48	700,000	2	30.43	4	5	3
	Horse 15	41.6	48	240,000	2	33.71	5	2	2
	Horse 20	41.4	48	52,500	2	31.32	7	2	3
Immune	Horse 6	38.3	12	ND	ND	ND	ND	93	993
	Horse 11	38.0	12	ND	ND	ND	ND	7	53
	Horse 14	38.1	12	ND	ND	ND	ND	27	234
	Horse 24	38.3	72	ND	ND	ND	ND	75	1,077

^*a*^ Immune status was determined following infection based on the presence of fever and viral detection.

^*b*^ Body temperatures were measured by rectal temperature; fever was defined as a body temperature of 38.6°C or higher.

^*c*^ Nasal shedding was measured in nasal secretions by plaque assay.

^*d*^ Cell-associated viremia was measured in PBMC by EHV-1-specific qPCR.

^*e*^ Intranasal EHV-1-specific IgG4/7 antibodies were measured by a fluorescent bead-based assay in nasal secretions pre-infection and at 24hpi.

^*f*^ ND = not detected; MFI = median fluorescence intensity; PFU = plaque-forming units

^*g*^ All horses were intranasally infected with 1 × 10^7^ PFU neuropathogenic EHV-1 Ab4 and monitored daily for clinical signs of disease.

### Differential gene expression between immune and non-immune horses early in EHV-1 infection

**Fig 2:**
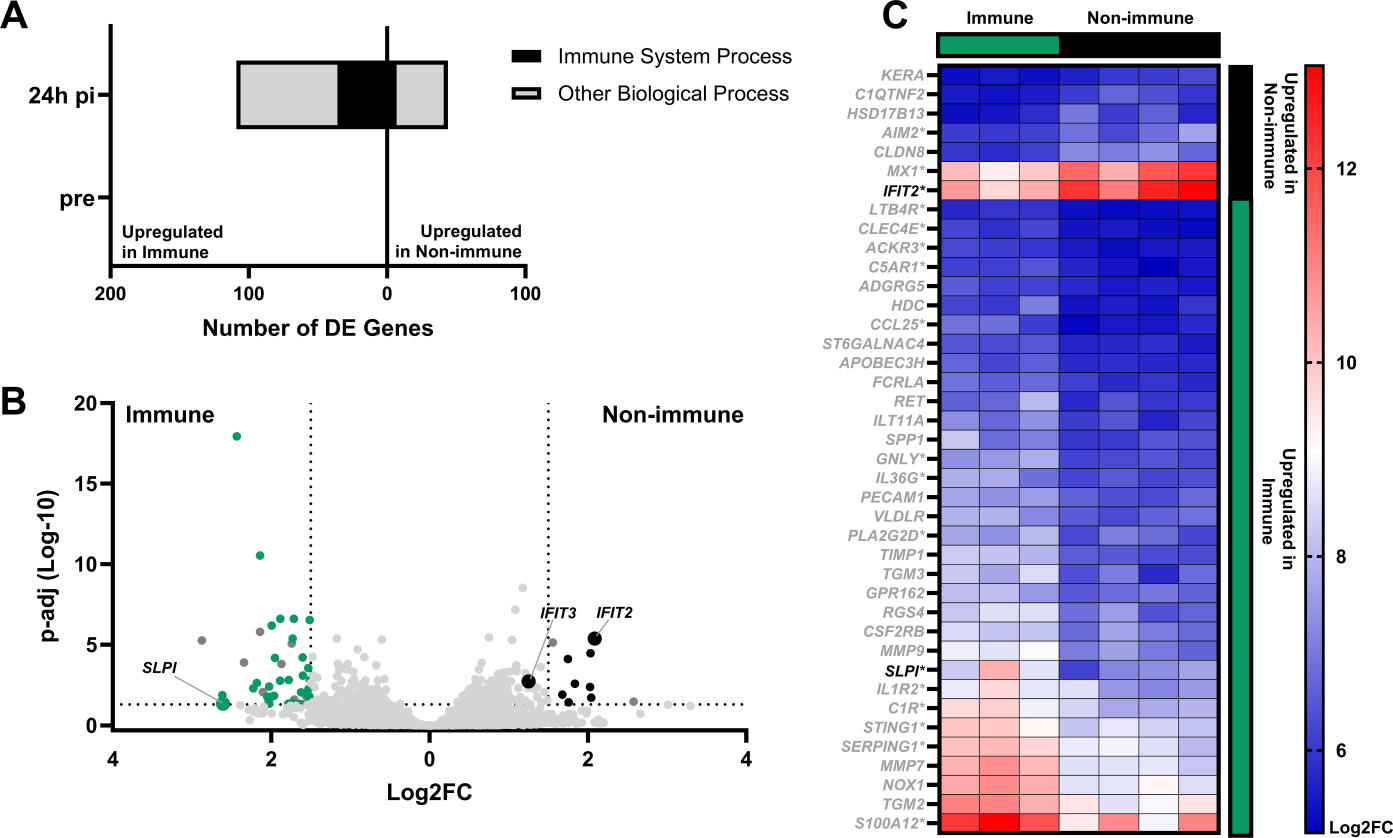
Differential expression of genes in non-immune compared to immune horses during early infection with EHV-1. RNAseq was used to quantify nasopharyngeal gene expression in EHV-1 immune and non-immune horses, pre-infection and at 24hpi. DE genes were determined by DESeq2, and gProfiler was used to determine the immune relevance of DE genes. (**A**) Number of DE genes between immune and non-immune horses, pre-infection (bottom) and at 24hpi (top) with genes upregulated in immune horses to the left and genes upregulated in non-immune horses to the right. The fraction of DE genes related to the immune response is shown in black, and genes involved in other biological processes are in gray. Genes were selected with a *P*-adj <0.10. (**B**) Volcano plot of group-specific LogFC against *P*-adj, dotted lines are the cut-off values for significant differential expression of Log2FC > 1.5 and *P*-adj <0.05. Green points represent annotated genes upregulated in immune horses, and black dots represent annotated genes upregulated in non-immune horses. Larger points with gene labels represent the selected gene for each group. (**C**) Heatmap with relative gene expression of each significantly upregulated gene by row. Columns under the green bar represent immune horses and columns under the black bar represent non-immune horses. Genes in black rows are upregulated in non-immune horses and genes in green rows are upregulated in immune horses. The intensity of shading represents relative expression in Log2FC for each horse. Selected genes are shown in black font. *Marks genes involved in the immune response.

PCA showed a rapid mucosal immune response, which was upregulated by 24hpi in response to respiratory EHV-1 infection. A differential gene expression analysis between immune and non-immune groups was conducted to find genes that were upregulated at 24hpi. A total of 153 DE genes were identified (Fig. 2A). Of the DE genes, 109 were upregulated in immune horses and the remaining 44 were upregulated in non-immune horses. There were no DE genes between groups before infection (Fig. 2A). The DE genes post-infection had a variety of biological functions. Upregulated genes both groups were involved in immune system processes, representing 33% and 16% of DE genes for immune and non-immune horses, respectively. To focus on the top DE genes for each group, a more stringent cut-off (Log2FC > 1.5 and *P*-adj <0.05) was applied, emphasizing genes with the greatest variation between groups (Fig. 2B). The genes with the greatest variation included immune response genes, as well as genes with other biological functions. Of these genes, 33 were upregulated in immune horses and seven were upregulated in non-immune horses (Fig. 2C). Two genes, one from each group, were selected for further analysis based on their high Log2FC, their relevance to the immune response and evidence from other species demonstrating anti-viral properties during respiratory infection. These genes were antileukoproteinase (*SLPI*) for the immune group and IFN-induced protein with tetratricopeptide repeats 2 (*IFIT2*) from the non-immune group. In addition, analysis for IFIT3 was included, due to its functional relevance in the IFIT2:IFIT3 dimer.

**Fig 3:**
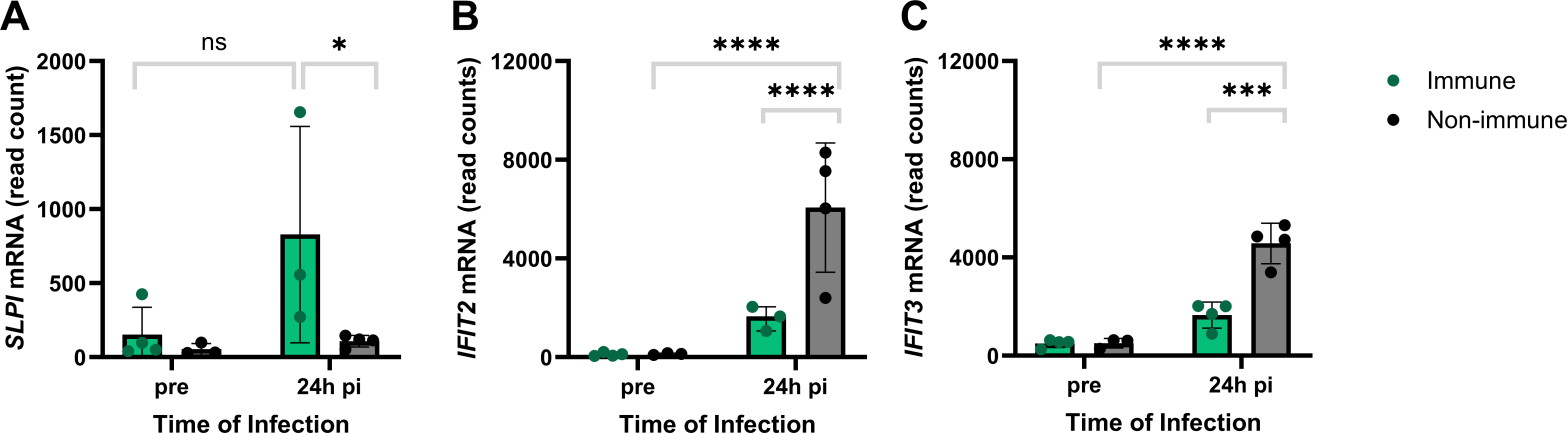
Gene expression of *SLPI*, *IFIT2,* and *IFIT3* differs in immune and non-immune horses at 24hpi. To compare the expression of the top genes, read counts for *SLPI, IFIT2,* and *IFIT3* were normalized and underwent variance stabilizing transformation. Immune horses are shown in green and non-immune horses are shown in black. (**A**) *SLPI*, (**B**) *IFIT2,* and (**C**) *IFIT3*. Bar graphs show the median and standard deviation and data points represent individual samples. ns = *P* > .05, *P < .05, ***P < .001, ****P < .0001.

At 24hpi, *SLPI* had 2.8-fold higher expression (*P*-adj = 0.0445) in immune compared to non-immune horses (Fig. 3A). Meanwhile, *IFIT2* was elevated in non-immune horses with 2.1-fold higher expression (*P*-adj <0.0001) compared to immune horses (Fig. 3B). Like *IFIT2*, *IFIT3* was elevated in non-immune horses, with 1.3-fold higher expression (*P* = 0.0018) compared to immune horses (Fig. 3C).

### Mucosal *SLPI* mRNA expression and protein secretion kinetics vary with EHV-1 immune status

**Fig 4:**
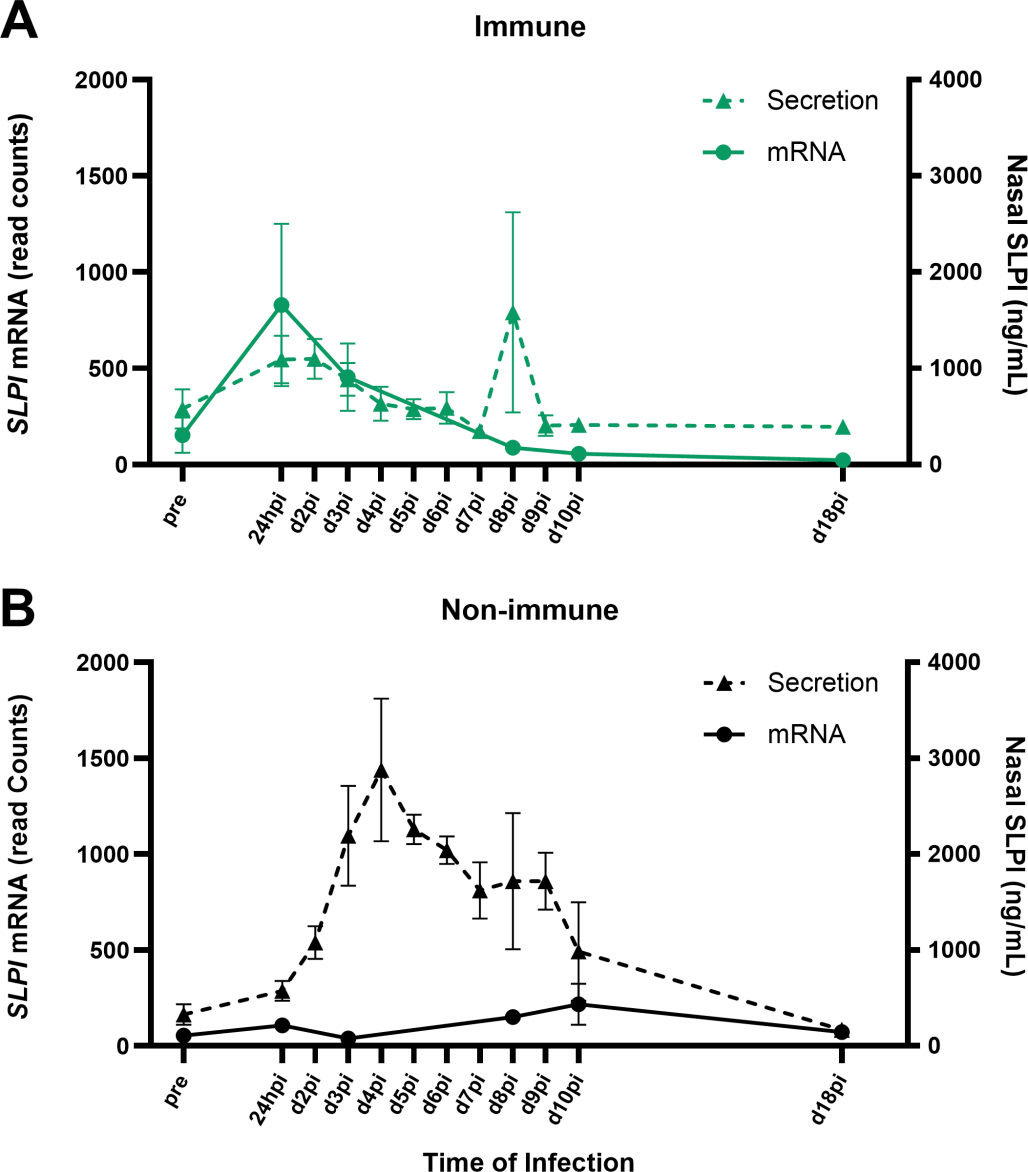
Mucosal *SLPI* mRNA expression in response to EHV-1 infection resembles protein secretion kinetics in immune horses. *SLPI* mRNA expression and protein secretion were measured at the URT and at additional timepoints in EHV-1 infection. *SLPI* mRNA was measured by RNAseq in nasal cells and protein secretion was measured by bead-based assay in the soluble fractions from the same nasopharyngeal swab samples. Immune horses are shown in green and non-immune horses are shown in black. (**A and **B) mRNA expression of *SLPI* compared to SLPI secretion in (A) immune horses and (B) non-immune horses. Solid lines indicate mRNA expression and dashed lines represent protein secretion. Graphs show the median and SEM.

To further explore the differential expression of *SLPI* after EHV-1 infection, RNA sequencing analysis of samples taken at additional timepoints, including days 3, 8, 10, and 18pi, supported that *SLPI* RNA expression peaked at 24hpi in immune horses, decreased afterwards, and returned to pre-infection values by d10pi (Fig. 4A). Next, SLPI mRNA expression and protein secretion were compared in nasal secretion samples. SLPI secretion in immune horses was similar to the mRNA expression profile (Fig. 4A). By contrast, *SLPI* mRNA expression in non-immune horses did not resemble protein secretion. While mRNA expression stayed almost constant throughout the EHV-1 infection, protein secretion increased after 24hpi, peaked at d4pi, and returned to baseline by d18pi (Fig. 4B).

**Fig 5:**
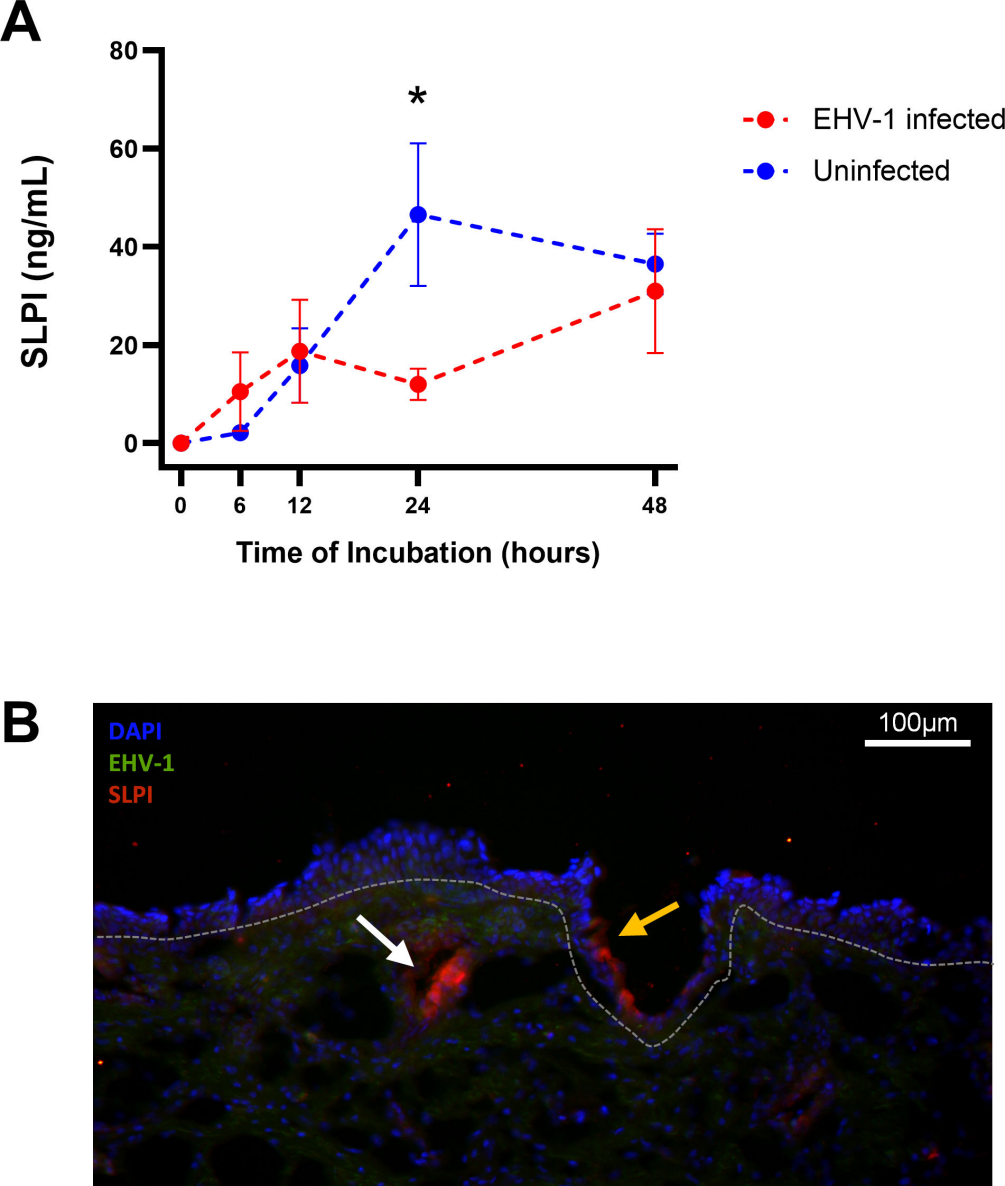
SLPI is produced by respiratory epithelial cells and cells beneath the basement membrane. To determine the localization of SLPI-producing cells, nasal explants were infected with EHV-1-GFP *in vitro*. Both supernatant and explant tissue were collected to determine SLPI secretion and the location of SLPI-producing cells within the nasal mucosa. (**A**) SLPI secretion in the supernatants of nasal explants was measured in a bead-based assay. Supernatants from uninfected (blue, *n* = 3) and EHV-1-infected tissue replicates (red, *n* = 3) were compared. Graphs display median and SEM. * Denotes *P*-value < 0.05. (**B**) Representative image of tissue expression for SLPI in EHV-1-infected explants after 24 hours of incubation. Nuclear staining for DAPI in blue, SLPI in red, and EHV-1-GFP in green. The basement membrane is denoted by the grey dotted line, the yellow arrow marks epithelial SLPI expression, and the white arrows mark vascular and glandular SLPI expression.

To evaluate the discrepancy in SLPI mRNA and protein expression in non-immune horses and to identify the location of SLPI-secreting cells in the URT, a nasal explant model was established. Nasal tissue was collected from a healthy horse and infected with EHV-1-GFP *in vitro*. Supernatants collected from control tissues indicated continual secretion of SLPI during the 48-hour culturing period (Fig. 5A). Infection of the nasal explant with EHV-1 resulted in a significant decrease (*P* = 0.036) in SLPI secretion at 24 h, compared to uninfected controls. In addition, immunofluorescence imaging was performed to determine the location of SLPI-producing cells in the nasal tissue. Within EHV-1-infected explants, some of the epithelial cells produced SLPI. In addition, glandular and vascular tissue beneath the basement membrane had high SLPI production (Fig. 5B). This supported that SLPI can be expressed and secreted by cells located deeper within the nasal mucosa.

### Mucosal *IFIT2* and *IFIT3* expression concurs with IFN-α secretion

**Fig 6:**
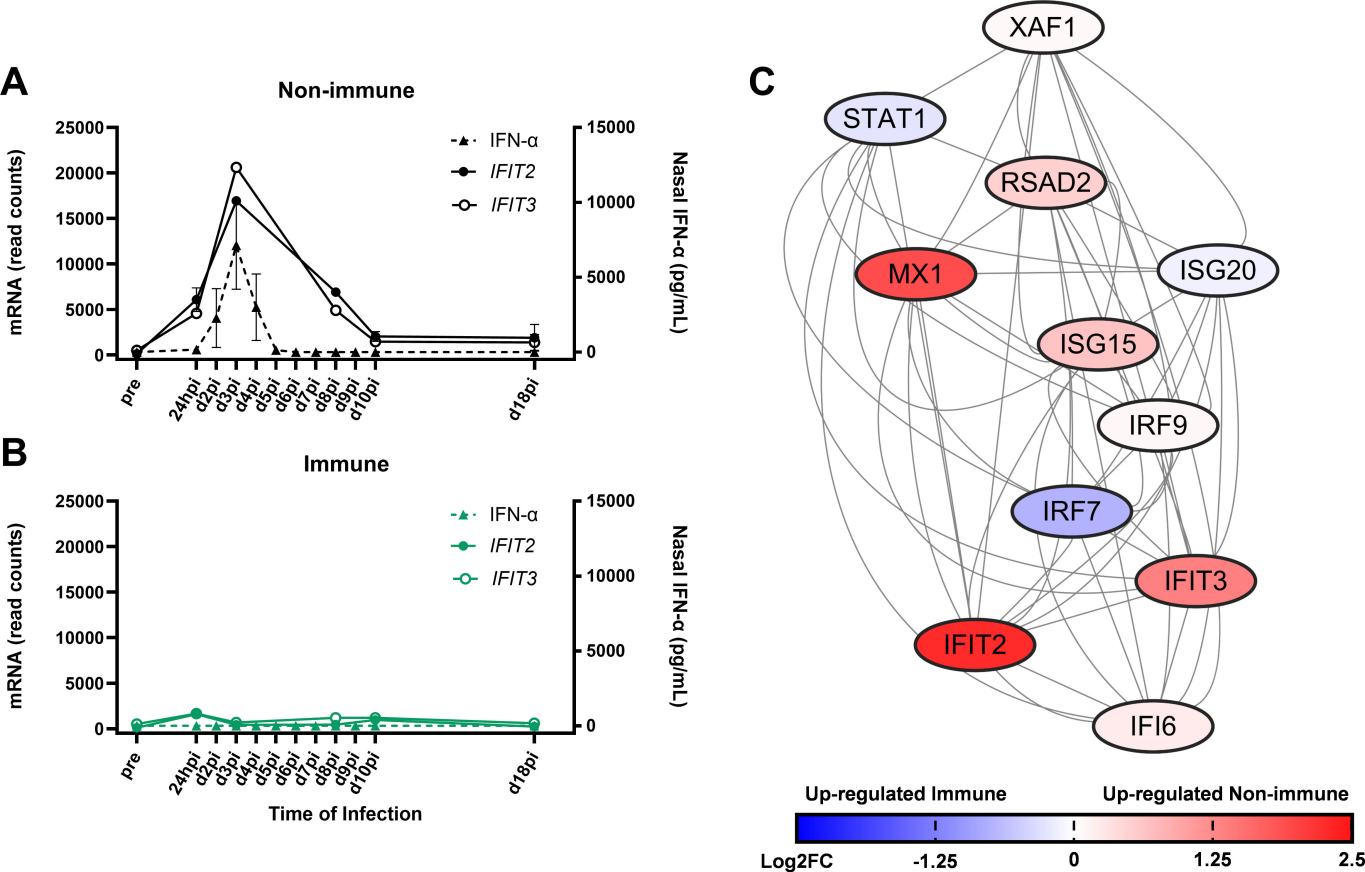
IFIT2 mRNA expression at the nasal mucosa corresponds with increased IFN-α secretion. *IFIT2* and *IFIT3* mRNA expression and IFN-α secretion were analyzed in samples taken from the same location at the URT. Immune horses (green) and non-immune horses (black) are compared throughout the EHV-1 challenge. (**A and B**) mRNA expression of *IFIT2* and *IFIT3* compared to IFN-α secretion in (A) non-immune horses and (B) immune horses with solid lines for mRNA expression and dashed lines for protein secretion. Graphs display median and SEM. (C) STRING network of known and predicted protein interactions with IFIT2 and IFIT3. Each node represents a protein and is labeled with the protein abbreviation. The intensity of color for each node corresponds to fold change of gene expression for that protein, with red representing higher expression in non-immune and blue representing higher expression in immune horses. Connecting lines represent protein-protein interactions, and node location from top to bottom represents hierarchal position, based on time of protein expression during a type I interferon response.

The *IFIT* gene family belongs to the group of interferon-stimulated genes that promote host anti-viral defense mechanisms. *IFIT2* and *IFIT3* were upregulated in non-immune horses at 24hpi. *IFIT2* and *IFIT3* expression peaked at d3pi and slowly decreased through the resolution of clinical disease by d10pi (Fig. 6A). Expression profiles of genes, *IFIT2* and *IFIT3*, were similar throughout the infection. Upregulation of *IFIT2* and *IFIT3* mRNA also aligned with the increased intranasal secretion of IFN-α in non-immune horses. Gene expression and protein secretion shared a peak on d3pi and continued expression of *IFIT2* and *IFIT3* was maintained, even after IFN-α became undetectable by d5pi (Fig. 6A). Immune horses did neither increase *IFIT* gene expression nor IFN-α secretion at the nasal mucosa after EHV-1 infection (Fig. 6B). IFIT2 and IFIT3 are cytosolic proteins that directly interact with other proteins in their local environment to inhibit viral replication. The STRING database was utilized to map a network of known and predicted protein interactions of equine IFIT2. The hierarchal STRING network demonstrated that genes of the proteins upstream of IFIT2 were also differentially expressed (DE) during early infection (Fig. 6C).

## DISCUSSION

EHV-1 immunity is considered of crucial importance in preventing severe disease outcomes and reducing viral transmission in the horse population (15, 16). A few studies on mucosal immunity at the viral entry site, the URT, identified antibody and cellular immune responses against EHV-1 in horses after clearing the virus and recovering from the disease (15, 16, 25, 28, 29). More recent work in horses with pre-existing EHV-1 immunity, that were experimentally challenged with EHV-1, has shown a rapid increase in mucosal virus-specific antibodies, together with limited local inflammation at the respiratory viral entry site. By contrast, non-immune horses lacked mucosal antibodies early after infection while secreting a wide variety of local inflammatory proteins in response to EHV-1 infection (15, 16, 28, 29). The early mucosal antibody responses to EHV-1 in immune horses highly correlated with protection from disease, including prevention of viral replication, transmission by nasal shedding, and cell-associated viremia. The findings support the hypothesis that pre-existing adaptive mucosal immunity has an essential role in orchestrating the early host immune response and defense against EHV-1 at the viral entry site.

**Fig 7:**
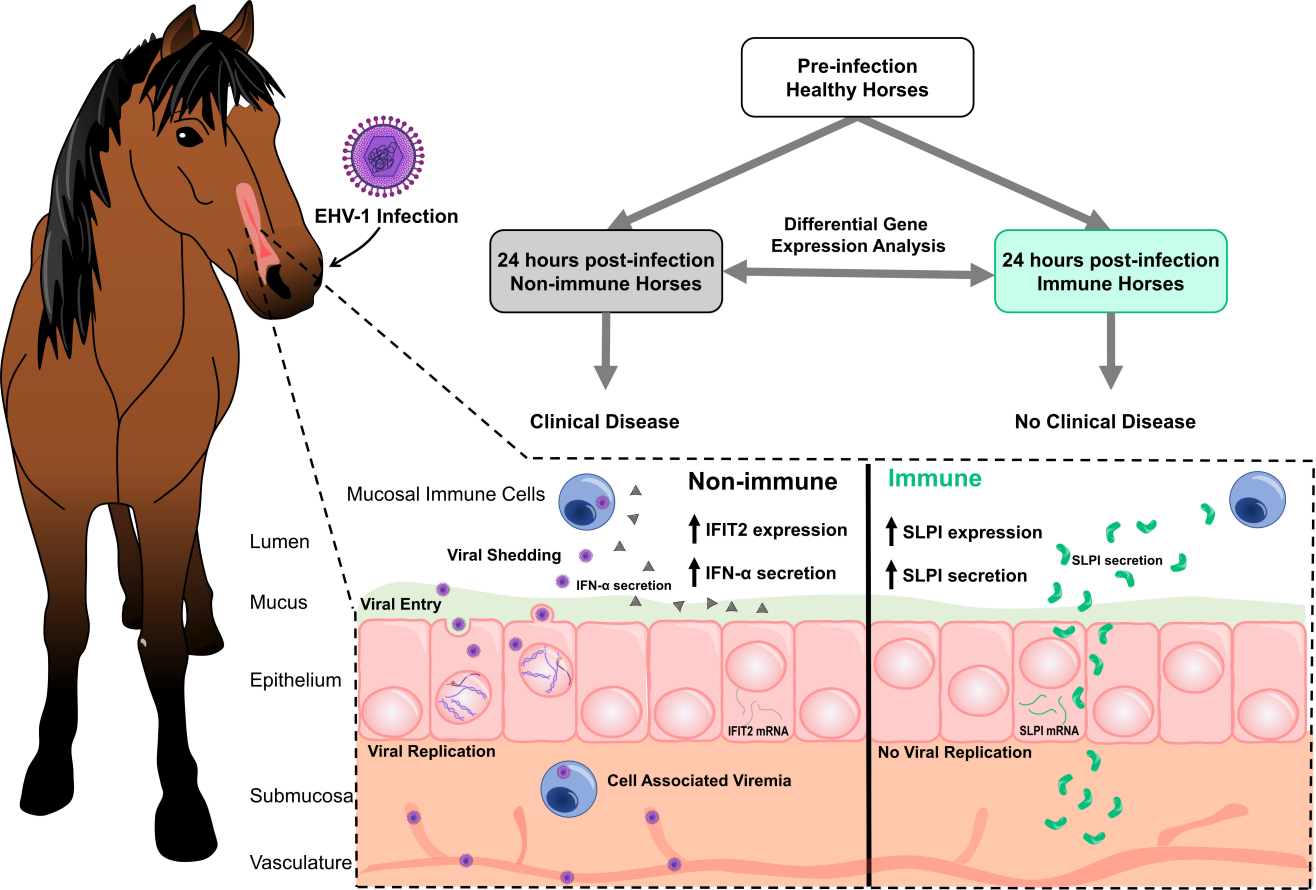
Immune horses increase SLPI while non-immune horses increase type I interferon secretion during the early mucosal innate immune response to EHV-1. RNAseq analysis of nasal samples unveiled rapid differential gene expression in immune (green) and non-immune (gray) horses within 24 hours of EHV-1 infection. Early differences in gene expression and corresponding protein concentrations were indicative of clinical outcomes and a lack of viral shedding and cell-associated viremia in immune horses. Immune horses increased expression and nasal concentration of SLPI (green boomerangs), while non-immune horses increased expression of ISGs and nasal concentration of IFN-α (gray triangles).

Here, we aimed to further characterize mucosal immune response activation by EHV-1 and to identify immune components and pathways that are shaped by the EHV-1 immune status of the host at the time of infection. Transcriptomic profiling was performed on banked nasopharyngeal swab samples of immune and non-immune horses, from a previously described experimental EHV-1 infection study with Ab4 (16). Group differences in gene expression at 24hpi identified a total of 153 differentially expressed genes with a range of functions, both in immune responses and other biological processes. This suggested that early changes in gene expression are occurring in immune and non-immune horses, with the virus eliciting an immune response from resident mucosal cells and local immune cells. Three of the immune response genes, *SLPI*, *IFIT2,* and *IFIT3*, were further analyzed in EHV-1 immune and non-immune horses, by identifying their mucosal gene expression at additional timepoints during EHV-1 infection, and by comparing gene expression findings with protein measurements in the same nasal secretion samples (Fig. 7). These top candidates are novel targets in the early mucosal immune response against EHV-1, with *IFIT2* and *IFIT3* representing a pathway involved in the type I interferon response, and *SLPI* representing a unique molecule not previously explored in equine anti-viral responses.

At 24hpi, *SLPI* gene expression was upregulated only in immune horses. SLPI is a pleiotropic molecule, which is secreted at mucosal surfaces and systemically (39, 40). It has three primary functions in the immune response as an upholder of homeostasis: inhibition of pathogens (41, 42), downregulation of inflammation (43–45), and inhibition of immune cell-derived serine proteases (39, 46). The role of SLPI in the mucosal immune response to viral infection has been most extensively explored in viruses of the female reproductive tract, including human immunodeficiency virus (HIV) (47), herpes simplex virus type 2 (HSV-2) (48–50), and human papillomavirus (HPV) (48, 51). During HIV and HPV exposure, SLPI can limit viral entry through inhibition of interactions with viral entry receptors, CD4 and annexin, respectively (47, 48, 51). Interestingly, HSV-2 can downregulate SLPI as a suspected immune escape mechanism (48–50). *In vitro,* reduction of SLPI occurred both at the transcript and protein level and required HSV-2 viral gene expression (49). Like HSV-2, EHV-1 is an alphaherpesvirus that infects mucosal epithelial cells, and both viruses share the same set of core proteins (52). Here we saw an early increase in *SLPI* gene expression and elevated SLPI secretion within 24hpi in immune horses that did not have detectable viral replication at the URT. Meanwhile, in non-immune horses, high amounts of replicating virus were detectable while mucosal *SLPI* gene expression did not increase. This suggests that successful EHV-1 entry and replication inhibited *SLPI* gene expression at the mucosal surface in non-immune horses, similar to the mechanism described above for HSV-2. The inhibition of SLPI by EHV-1 was further supported by decreased SLPI secretion from EHV-1-infected nasal explant tissues. In contrast to immune horses, high amounts of mucosal antibodies are present within 24hpi (15, 16) which likely contributes to immediate virus neutralization resulting in the observed decrease in virus replication. Together, this suggests that effective EHV-1 neutralization enables the synchronized early gene expression and secretion of SLPI in immune horses. This points to an essential role of SLPI in early immunoregulation after EHV-1 exposure, by orchestrating the mucosal immune response toward protection from disease after infection and return to homeostasis.

While non-immune horses did not upregulate nasal *SLPI* gene expression, the protein was detectable in the nasal secretions at high concentrations, peaking on d4pi. The delayed onset of SLPI secretion occurred when the replicating virus decreased at the URT of non-immune horses, and high mucosal SLPI concentrations were maintained for several days while clinical signs of disease were present. The discrepancy between RNA expression and protein production can be impacted by translation rates, modulation of translation, protein half-life, protein synthesis delay, and or protein transport (53). Out of these, only protein transport would explain lower transcript expression than protein secretion. In fact, EHV-1 infection of nasal explant tissue *in vitro* supported the existence of SLPI-producing cells beneath the basement membrane, agreeing with previous characterization of SLPI in equine URT tissue (40). While SLPI was induced and transported to the nasal cavity upon EHV-1 infection, these deeper tissue cells were not captured by the nasopharyngeal swab sample that was used for the transcriptome analysis. Additional work would be needed to further characterize the cell types contributing to nasal SLPI secretion. Taken together, productive EHV-1 infection in non-immune horses inhibited mucosal *SLPI* gene expression, while SLPI secretion was delayed and likely originated from cells in the underlying tissues, which are not directly interacting with the virus during early infection.

Non-immune horses respond to EHV-1 infection by upregulating interferon-stimulated genes *IFIT2* and *IFIT3* in concert with IFN-α secretion until the virus is cleared from the URT. In most species, the IFIT gene family consists of four members IFIT1, IFIT2, IFIT3, and IFIT5, which in humans can work alone or in complex (54, 55). Here, we saw co-expression of *IFIT2* and *IFIT3* supporting the preferential formation of the IFIT2:IFIT3 heterodimer (55). This gene family is conserved across the majority of mammalian species, apart from mice and horses, which lack IFIT5 and IFIT1, respectively (56). Functional differences in the equine IFIT proteins and the implications of the unique loss of IFIT1 in horses have not yet been explored. However, IFIT2 and IFIT3 have a range of roles in the anti-viral response. Their high expression early during EHV-1 infection in non-immune horses suggests they are essential in the innate immune response initiation and anti-viral defense. However, *IFIT2, IFIT3,* and IFN-α were not increased in immune horses at any time after infection. Together, this demonstrated that the early secretion of mucosal IFN-α in non-immune horses promotes the upregulation of interferon-stimulated genes, including *IFIT2* and *IFIT3*, while activation of this pathway seems unnecessary for EHV-1 immune horses to control and clear infection.

In humans and mice, IFIT2 acts as an apoptotic factor through activation of the caspase-3 pathway (55, 57), as an anti-inflammatory regulator through binding host-derived transcripts (58, 59), or directly as an anti-viral factor through viral nucleic acid binding (54, 55). IFIT2 has a demonstrated role in inhibition of viral replication which was characterized in mouse models of neurotropic viruses. *In vitro* depletion of murine IFIT2 led to increased replication of West Nile Virus (WNV) and Rabies virus in neurons (60, 61). Similarly, *IFIT2*^-/-^ mice have increased viral replication *in vivo* during vesicular stomatitis virus, WNV, and mouse hepatitis virus (strain A59) infection (60, 62–64). In all cases, mice-expressing *IFIT2* had decreased neuropathology, with lessened or absent viral replication in the neurons and/or brain. IFIT2 inhibition of viral replication in neural cells may also be of importance for EHV-1, which can establish latency in the trigeminal ganglia of sensory neurons (65). Based on these findings, it can be hypothesized that high *IFIT2* expression can lead to prevention from neurologic EHM after EHV-1 infection of naïve or non-immune horses. However, this correlation still needs further experimental evidence in the future EHV-1 work.

In human viral infection, IFIT3 has roles in interrupting herpesvirus replication (66), inducing cell cycle arrest through sequestration of Jun activation domain-binding protein 1 (JAB1) (67), or promoting the type 1 IFN response by facilitating protein interactions (68, 69). Herpes simplex virus type I (HSV-1) is capable of virally mediated degradation of *IFIT3* transcripts *in vitro* through RNase action of UL41. However, in a mouse UL41 knockout strain of HSV-1 (R2621), unimpeded IFIT3 expression reduced viral replication (66). While EHV-1 encodes for a UL41 homolog (52), we saw the expression of *IFIT3*, suggesting EHV-1 does not possess the same immune escape mechanism. This may allow IFIT3 to act in an inhibitory role during viral replication, as was described in R2621. In addition, previous work has demonstrated a robust type I interferon response in EHV-1 susceptible- (non-immune) horses (16, 28, 70, 71). Here, *IFIT3* expression was increased 24 hours before IFN-α secretion began, suggesting a role in promoting the type I IFN response. Overall, high expression of *IFIT3* supports an innate anti-viral role during EHV-1 infection, with additional experiments needed to determine the exact mechanism.

### Conclusions

In summary, transcriptomic profiling served as a tool for the initial identification of new targets in the mucosal immune response to EHV-1 infection. We have characterized three new immune system genes orchestrating the early mucosal immune response to EHV-1. *IFIT2* and *IFIT3* gene products work to promote the antiviral state, and in turn, SLPI is a homeostatic regulator preventing an overzealous response. Non-immune horses activated all three of these genes or proteins at different stages of EHV-1 infection to first initiate and then control the anti-viral immune response. By contrast, immune horses were able to rapidly clear the virus largely independent of inflammation and in the absence of mucosal *IFIT2* and *IFIT3* gene upregulation and IFN-α secretion. By 24hpi, immune horses upregulated SLPI to promote the early return to homeostasis in EHV-1-protected horses. Overall, these data highlight the efficiency of the early mucosal immune response at the site of viral entry in preventing viral replication and disease.

## MATERIALS AND METHODS

### EHV-1 infection history of the horses

All samples for RNA sequencing were collected during a prior experimental EHV-1 infection and were banked at −80°C for future analysis based on immunity. The original study was described previously in detail (16). Samples for this approach were obtained from adult Icelandic horses 3 to 4 years of age, including one mare and three geldings per group. The immune status of the horses as immune or non-immune was determined retrospectively, after analyzing all viral and clinical parameters. Non-immune horses (*n* = 4) developed a fever after EHV-1 infection, shed high amounts of virus in their nasal secretions, and developed cell-associated viremia (Table 1). Non-immune horses were last infected with EHV-1 two or more years prior to the current infection when the samples were taken for this study. Immune horses (*n* = 4) had no fever, and neither infectious virus nor cell-associated viremia were detected in their nasal secretions and peripheral blood cells, respectively. Immune horses also had mucosal and systemic EHV-1-specific IgG4/7 antibodies prior to infection and their mucosal IgG4/7 values rapidly increased by 24 hours post-infection (24hpi) (Table 1). Immune horses were infected with EHV-1 nine months before the current infection when samples were taken for this study. Pre-infection EHV-1-specific antibodies were missing in non-immune horses and antibodies stayed low on d1pi (27).

All animal procedures were approved by the Institutional Animal Care and Use Committee at Cornell University (protocol #2011-0011). Experimental EHV-1 infections and all sample collections were carried out in accordance with the recommendations in the Guide for the Care and Use of Laboratory Animals of the National Institute of Health and Animals in Agricultural Research and Teaching. All efforts were made to minimize the discomfort of the animals during procedures, for example by short sedation. After the end of this experimental study, all horses were kept at the facility at Cornell University as research horses.

### Nasopharyngeal swab samples

Nasopharyngeal swab samples were collected from horses as previously described (29). A polyester double-tipped applicator (Puritan Medical Products Company, Gullford, Maine, USA) was inserted into the nose and swabbed against the mucosa for 5 seconds. For RNA extraction, one swab from one nostril was stored in a dry tube, without any further additives, at −80°C until processing. For protein detection, the other swab was placed in a tube with 1 mL phosphate-buffered saline (PBS, 137 mM NaCl, 2.7 mM KCl, 4.3 mM Na2HPO). Tubes were vortexed for 15 s and centrifuged at 900 × *g* to separate large debris. The upper aqueous phase was collected and transferred to a new tube, which was centrifuged at 300 × *g* to remove the remaining particles and cells. The upper aqueous phase was aliquoted into a new sterile tube and stored at −20°C until further analysis. Samples for RNAseq were taken from eight horses on d-2 and 24hpi, d3pi, d8pi, d10pi, and d18pi. Samples for protein detection were taken from the same eight horses on d-2, daily from 24hpi to d10pi, and d18pi.

### RNA extraction

For RNA extraction, nasopharyngeal swabs were reconstituted in 800 µL MagMAX Lysis/Binding Solution and 250 µL of the sample was utilized for RNA sequencing (Ambion Inc, Austin, TX, USA). RNA was extracted using TRIzol LS reagent (Life Technologies Corporation, Carlsbad, CA, USA) following the manufacturer’s protocol. RNA sample quality was confirmed by spectrophotometry to determine the concentration and chemical purity (A260/230 and A260/280 ratios) and with a Fragment Analyzer (Advanced Analytical, Orangeburg, NY, USA) to determine RNA integrity. Quality control checks were utilized to exclude one sample, which had low host gene expression. The remaining samples offered coverage of both the non-immune and immune groups during early viral infection.

### RNA sequencing

RNA samples from immune (*n* = 4) and non-immune (*n* = 4) horses were submitted to Cornell University’s Transcriptional Regulation and Expression (TREx) facility for sequencing. Ribosomal RNA was subtracted by hybridization from total RNA samples using the NEBNext rRNA Depletion Kit (Human/Mouse/Rat v2, New England Biolabs, Ipswich, MA, USA). RNA was processed using NEBNext Ultra II RNA Library Prep Kit (New England Biolabs, Ipswich, MA, USA) to create a cDNA library, which was quantified with a Qubit 2.0 (dsDNA HS Kit, Thermo Fisher, Waltham, MA, USA), and fragment size distribution was determined by Fragment Analyzer (Advanced Analytical, Orangeburg, NY, USA). Samples were sequenced on a Nextseq 500 at Cornell’s Biotech Core Facility, with at least 10M read generated per library. Reads were trimmed for loq quality and adaptor sequences using TrimGalor v0.6.0, cutadapt, and fastQC. Sequences were aligned to the horse genome, version EquCab 2.0 using STAR v2.7.0e, to generate a raw counts table.

### RNAseq analysis

Sequence data were analyzed using integrated Differential Expression and Pathway analysis (iDEP.951), as previously described (72). Raw read counts were uploaded and annotated with the equine genome assembly (EquCab3.0). Counts were normalized using a variance stabilizing transformation (VST). A PCA (73), for the first (PC1) and second (PC2) principal components, was used to demonstrate variation among the samples, and a pathway analysis of the PCA rotation was used to determine pathways upregulated based on grouping. PC1 explains the highest degree of variation within the samples, and PC2 represents the next level of variation following the removal of the variation described by PC1. Differential gene expression analysis based on the negative binomial distribution (DEseq2) (74) was used to determine DE genes, based on the combination of immune status and/or timepoint. The cut-off value for significance was set at false discovery rate (*P*-adj) <0.10 and for fold change was set at log2FC < ±1.5. The distribution of all genes in the data set was visualized in a volcano plot, graphing log2FC by *P*-adj. Significant differentially expressed genes were visualized in a heatmap, with relative log2FC of individual samples represented by the intensity of shading. For the selected genes, the normalized read counts, based on VST transformation, were graphed. Individual points represent individual samples, and statistics were determined by Deseq2.

To determine whether DE genes were functionally involved in the immune response, g:Profiler (version e104_eg51_p15_3922dba) was used (75). The equine genome assembly (EquCab3.0, GenBank accession: GCA_002863925.1) was used for reference DE genes; within the Gene Ontology biological process (GO:BP) term, “immune system processes” (GO:00002376) were determined.

### STRING network

The Search Tool for the Retrieval of Interacting Genes/Proteins (STRING) database (76) was utilized to generate a network of protein-protein interactions for IFIT2. This database makes use of both known interactions found in published data and predicted interactions based on neighborhood, fusion-fission events, occurrence, and/or co-expression. The *Equus caballus* reference (NCBI taxonomy ID: 9796) was used, and IFIT2 was searched. A high confidence score of 0.900 was selected, and the top 10 interactors were mapped. The lowest score for the top interactors was 0.971, with all genes selected based on the combination of co-expression, text mining of published work, and database entries. The resulting network was exported and customized in Cytoscape (version 3.9.1) (77), to include expression data found in the data described here. Each node represents a gene containing the gene abbreviation. The color and intensity of each node represent Log2FC for that gene, with red representing upregulation in non-immune horses and blue representing upregulation in immune horses. Genes were arranged hierarchically, with each edge/connecting line representing a protein-protein interaction.

### Quantification of nasally secreted proteins

IFN-α and SLPI concentrations were quantified in nasal secretion samples, using fluorescent bead-based assays. IFN-α was measured in undiluted samples by equine cytokine multiplex assay as previously described (78). SLPI was measured by a fluorescent bead-based assay, as recently described (40). Nasal secretion samples were applied at a 1:25 dilution to the SLPI assay, according to the previously described optimization. All other steps and assay components were the same as previously described (40, 78). The assays were measured in a Bio-Plex 200 instrument (Luminex, Austin, TX USA).

### Nasal explants

Nasal mucosal tissue was collected from the ventral nasal concha of both the right and left nostrils. The donor horse was a 4-year-old adult Thoroughbred gelding, with a history of equine protozoal myeloencephalitis, and was euthanized due to hind end weakness related to this condition. Tissue sections were washed in cold PBS containing antibiotic-antimycotic (300 units/mL of penicillin, 300 µg/mL of streptomycin, amphotericin B 0.75 µg/mL, Gibco). The underlying connective tissue was removed by dissection and the remaining mucosal tissue was divided into 24 equal pieces, using a 6-mm biopsy punch (Miltex, York, PA, USA), to allow for three replicates for each condition at four timepoints. The mucosal explants were then placed into a 24-well plate containing cell culture media [DMEM supplemented with 1% (vol/vol)] non-essential amino acids, 2 mM L-glutamine, 50 µM 2-mercaptoethanol, 50 µg/mL gentamicin, 100 U/mL penicillin, 100 µg/mL streptomycin (Thermo Fisher Scientific, Waltham, MA, USA), and 10% FCS (Atlanta biological, Flowery Branch, GA, USA). The explants were suspended on sterilized gauze to generate an air-liquid interface and rested overnight. The next morning half of the explants were inoculated for 1 hour with 1 mL of EHV-1 Ab4-GFP (79) at 4 × 10^5^ Pfu/mL. Then, infected explants were washed twice with warm cell culture media and all explants were transferred to a new plate with fresh cell culture media. Tissue explants and supernatant were collected from three wells for each condition at four timepoints post-inoculation: 6, 12, 24, and 48 hours. The supernatant was aliquoted and stored at −20°C until quantification in the SLPI bead-based assay. Tissues were washed in 5 mL PBS and frozen at −80°C in Optimal Cutting Temperature compound (Tissue-Tek OCT, Sakura Finetek USA, Inc. Torrance, CA USA) until visualization by immunofluorescence.

### Immunofluorescence of nasal explants

Slides were cut from nasal explant tissue blocks using a cryostat. Ten 10 µm sections were cut from each block and adhered to charged glass slides. To prepare for staining, tissue was fixed in 10% (vol/vol) formalin solution (VWR, Radnor, PA USA) for 10 minutes and washed twice in deionized (DI) water for 10 minutes at room temperature. Tissue was blocked with 10% donkey serum (Equitech-Bio Inc, Kerrville TX USA) in PBST [PBS supplemented with 0.1% Tween 20 (VWR, Radnor, PA USA)], for 1 hour. Tissues were stained overnight at 4°C with SLPI mAb clone 27–1 at 10 µg/mL in 10% donkey serum in PBST. The stain solution was removed, and the slides were washed twice with PBST for 10 minutes. A secondary donkey anti-mouse IgG(H + L) antibody conjugated to Alexa-Fluor 594 (Invitrogen, Waltham, MA USA) was used for detection, diluted at 1:500 in 10% donkey serum in PBST, for 1 hour. The solution was removed, and the slides were washed for 10 minutes in PBST, followed by 3 minutes in DI water. Slides were mounted using Fluoroshield with DAPI (Sigma-Aldrich, St. Louis, MO USA) and stored in the dark at 4°C until visualization on a fluorescent microscope. SLPI was visualized at 595 nm, EHV-1 was visualized at 488 nm, and DAPI was visualized at 359 nm.

### Statistical analysis

Statistical analysis for explant SLPI secretion was performed using GraphPad Prism software version 8 (GraphPad Software, La Jolla, CA). An ordinary two-way ANOVA with Šídák’s multiple comparisons test was performed. SLPI secretion at each timepoint was compared for uninfected and EHV-1-infected nasal explants. Significance was set at alpha 0.05.

## Data Availability

The data sets generated and analyzed in this study are available in the NCBI’s Gene Expression Omnibus (GEO) https://www.ncbi.nlm.nih.gov/geo/query/acc.cgi?acc=GSE232941.
